# Genetic Risk of Rheumatoid Arthritis: A Case Control Study

**DOI:** 10.1007/s10528-023-10648-7

**Published:** 2023-12-30

**Authors:** Hosam M. Ahmad, Zaki M. Zaki, Asmaa S. Mohamed, Amr E. Ahmed

**Affiliations:** 1 Biotechnology and Life Sciences Department, Faculty of Post Graduate Studies for Advanced Sciences, Beni-Suef university, Beni-Suef, Egypt; 2 Internal Medicine and Biomedical Chemistry Departments, Egypt Ministry of Health and Population, Minia, 61511 Egypt; 3 Clinical Pathology Department, Faculty of Medicine, Minia University, Minia, Egypt; 4 Clinical Pharmacy and Pharmacy Practice Department, Faculty of Pharmacy, Port Said University, Port Said, Egypt

**Keywords:** Rheumatoid arthritis, ApaI, BsmI, Vitamin D, Calcium

## Abstract

Vitamin D effects are mediated by vitamin D receptors (VDRs), which are influenced by various genetic polymorphisms, including ApaI and BsmI. These polymorphisms have been linked to several diseases, including rheumatoid arthritis (RA). This study aimed to compare the frequency and association of VDR ApaI and BsmI gene polymorphisms, serum 25-hydroxy vitamin D (25-(OH)-D) levels, and calcium (Ca) levels between a RA group and a matched healthy control group. In one hundred RA patients and fifty healthy controls, the genotypes of the VDR ApaI and BsmI gene polymorphisms were analyzed using polymerase chain reaction restriction fragment length polymorphisms (PCR-RFLP). Both Serum 25-(OH)-D level and calcium level were measured in the two groups. There was no significant difference between the cases and controls regarding the VDR ApaI gene polymorphism (p = 0.89). A significant difference was observed between the cases and controls in terms of the VDR BsmI gene polymorphism (p = < 0.001). The serum levels of 25-(OH)-D and calcium were significantly lower in the RA group compared to the control group (p = 0.04 and < 0.001 respectively). Significantly higher serum vitamin D levels were associated with the aa genotype (p = 0.007). Significantly increased calcium levels were associated with the AA genotype (p = 0.02). No significant difference was found among BsmI polymorphisms regarding vitamin D and Ca levels (p = 0.25 and 0.87 respectively). Vitamin D receptor gene BsmI polymorphism but not ApaI polymorphism could be a marker of RA susceptibility. Vitamin D and Ca levels are negatively affected by RA. Vitamin D receptor gene ApaI polymorphism contributes to vitamin D and Ca levels.

## Introduction

Rheumatoid arthritis is defined as “an autoimmune disease that affects the joints and various body systems”. So far, the exact cause of the disease is not clear, but there are factors that can play an important role in the occurrence of the disease, including genetics, hormones, and environmental factors (Mohammed et al. 2021).

In spite of the remarkable medical progress in treating RA, a lot of patients still suffered from work incapacity and co-morbidities. Therefore, it is critical to quickly identify new potential risks to help identify and treat the disease early on. There is a an inherited component to the risk of RA (Perricone et al. 2011).

**Fig. 1:**
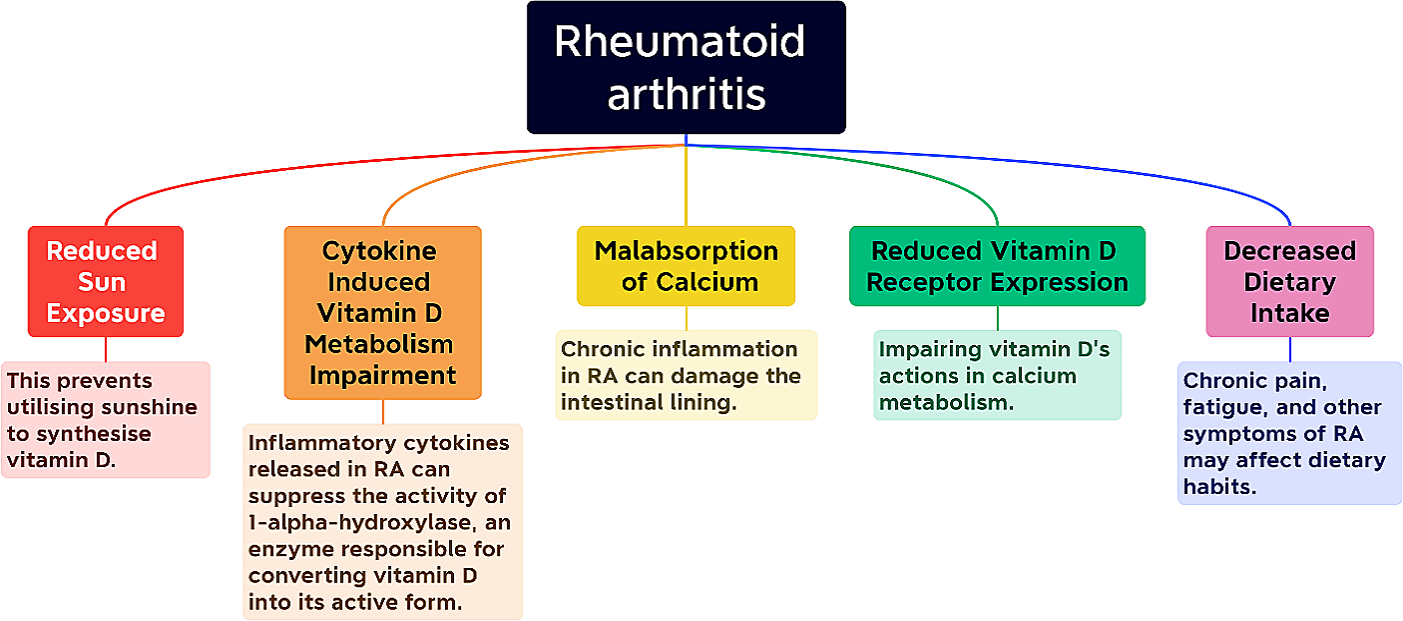
Mechanisms that RA can affect Ca and Vitamin D levels

Many researches revealed decreased calcium and/or vitamin D levels in rheumatoid arthritis patients by many mechanisms (Fig. 1) (Albedri Khudair 2020; Jambale and Halyal 2017; Watad et al. 2017).

Vitamin D is an immunoregulatory hormone. It is essential for maintaining the proper calcification of bone. A prior meta-analysis revealed a link between low vitamin D intake and an increased risk of RA (Song et al. 2012).

Increased vitamin D intake was linked to a reduced chance of developing RA. Furthermore, clinical improvement exhibited a strong association with the immunomodulating effects in vitamin D-treated RA patients (Mohammed et al. 2021). Vitamin D causes cellular responses by binding to the vitamin D receptor (VDR) (Haussler et al. 2013).

Nearly all tissues required for the actions of vitamin D have an active vitamin D receptor. The VDR gene has been found to have multiple genetic variants, including TaqI, BsmI, ApaI, and FokI (Gnagnarella et al. 2020).

Even while VDR polymorphisms have been linked to an increased risk of RA in multiple studies, the findings are still unclear. Moreover, VDR expression connected to ethnicity (O′ Neill et al. 2013) and has an impact on the genetic correlations in RA (Ghelani et al. 2011).

The present study aimed mainly to compare between RA patients and matched healthy controls regarding the frequency and the association of VDR ApaI and BsmI gene polymorphisms, serum 25-hydroxy vitamin D (25-(OH)-D) levels, and calcium (Ca) levels.

## Methods

### Study design

This study is a case-control study conducted at specialized rheumatology clinics.

### Study Participants

**Fig. 2:**
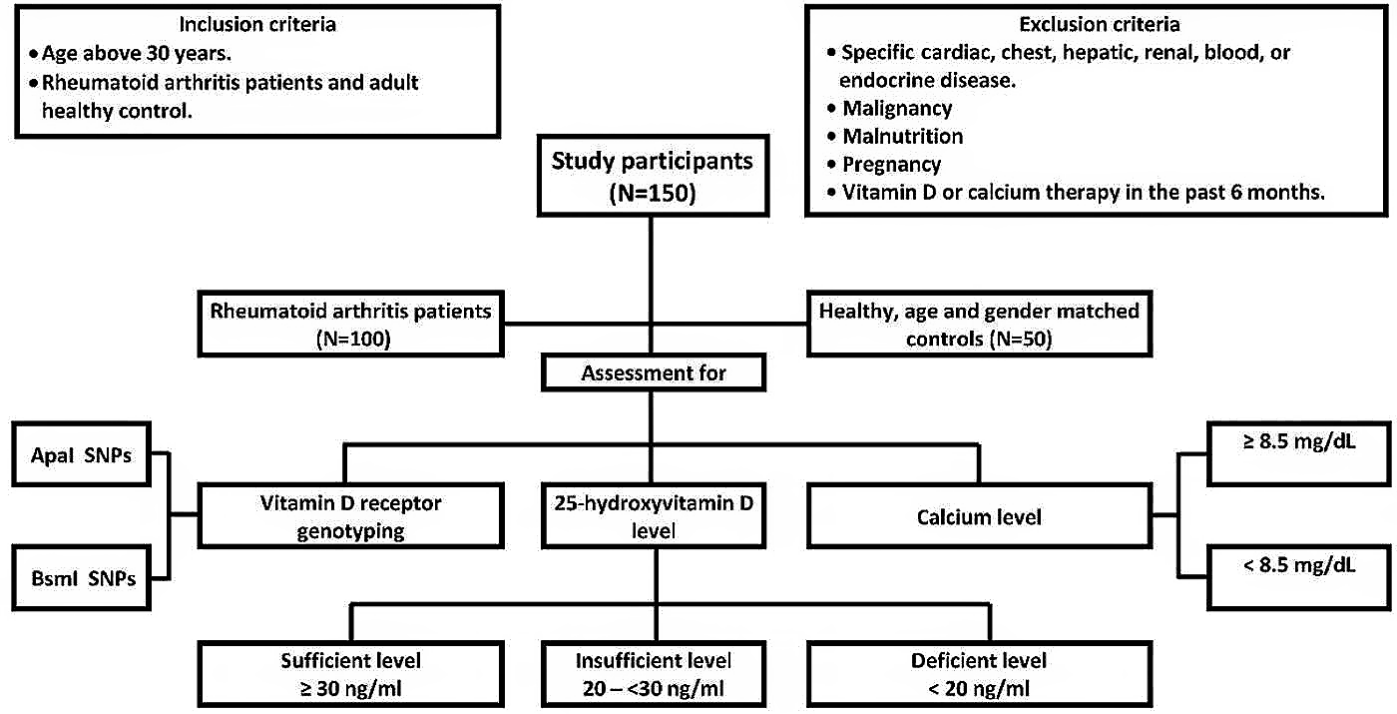
The main study steps

Two groups of adults (over 30 years) were enrolled. One hundred patients with prior diagnoses of rheumatoid arthritis made up the first group (cases), they fulfilled criteria for RA (Aletaha et al. 2010). Fifty healthy people who matched patients in terms of age and sex made up the second group (controls). The enrollment process was carried out in compliance with inclusion and exclusion criteria (Fig. 2).

### Procedures

The study sample for cases and controls groups was selected by consecutive sampling. Every participant underwent a history, examination, and medical investigations. The study steps were briefly explained in Fig. 2.

### Outcomes

For all study participants, we measured serum 25-hydroxy vitamin D (25-(OH)-D), calcium (Ca), and assessed them for VDR gene ApaI and BsmI polymorphisms. The 25-(OH)-Vitamin-D (TR) ELISA Kit was used to measure vitamin D3 in serum. The test principle is based on the competitive inhibition ELISA technique. Vitamin D levels were categorized into sufficient (≥ 30 ng/ml), insufficient (20 - < 30 ng/ml), and deficient (< 20 ng/ml). The amount of intact PTH in the serum was quantified using the Calbiotech ELISA Kit. Quantitative determination of calcium by spinreact o-cresolphtalein colorimetric kit. The level of calcium in the sample is based on the formation of a color complex between calcium and o-cresolphtalein in an alkaline medium. Calcium level normal range is (8.5–10.5 mg/ dL), it was categorized into (≥ 8.5 mg/dL), and (< 8.5 mg/dL).

### Genotyping

**Fig. 3:**
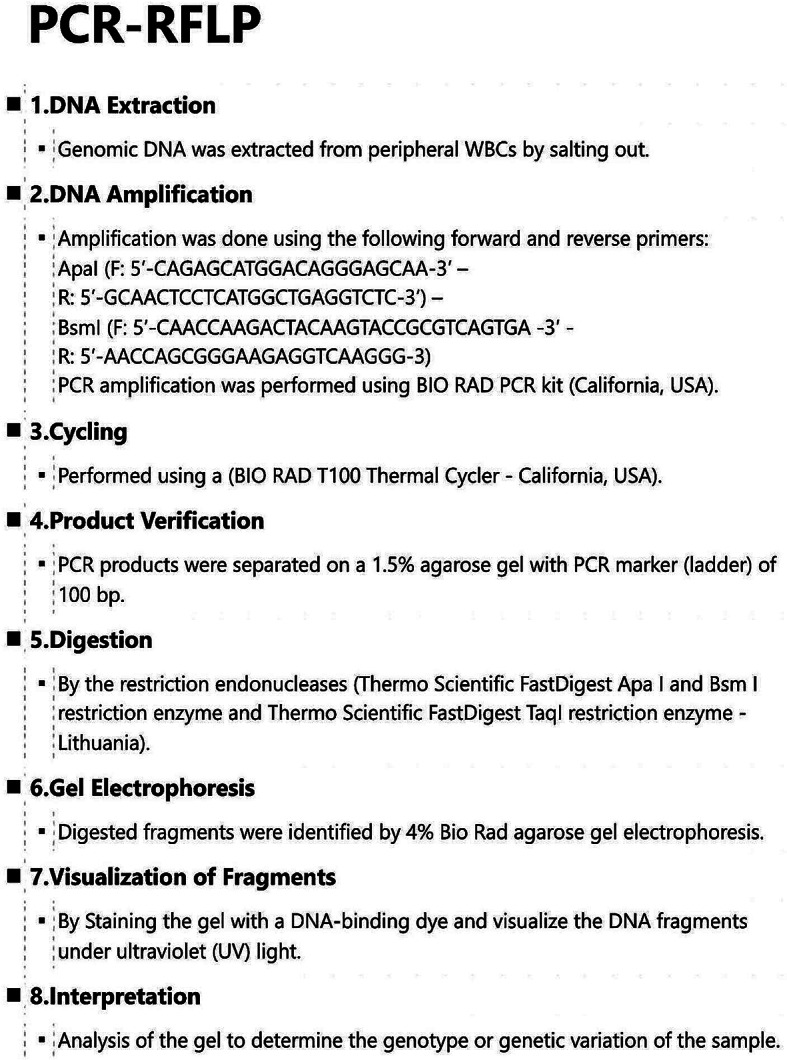
Determination of genotypes for VDR– ApaI and BsmI polymorphism by using PCR-RFLP method

The DNA was amplified using Polymerase Chain Reaction (PCR) and analyzed using the Restriction Fragment Length Polymorphism (RFLP) technique after digestion with specific restriction enzymes (Fig. 3).

**Fig. 4:**
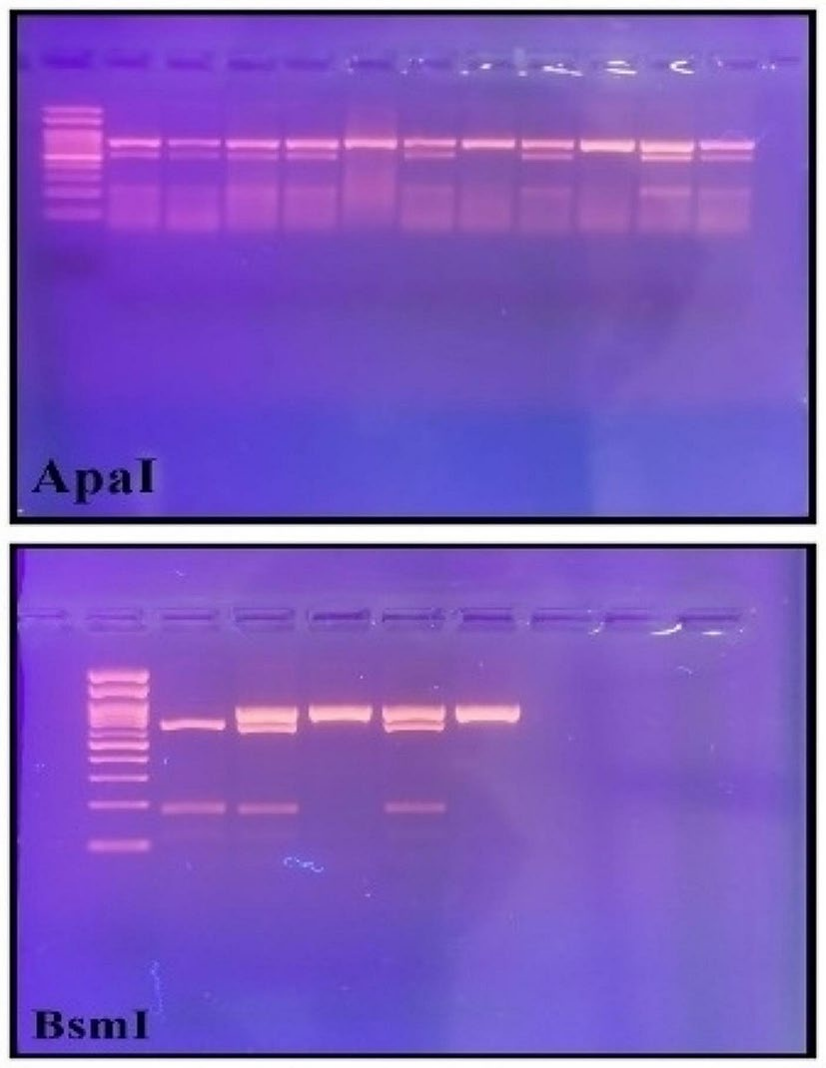
Determination of genotypes for VDR– ApaI and BsmI polymorphism by using PCR RFLP method

The size of the digested PCR products was as follows: For ApaI: The AA genotype led to one band at 740 bp, the aa genotype led to two bands at 530 bp and 210 bp, and the Aa genotype led to three bands at 740, 530, and 210 bp (Fig. 4). For BsmI: The BB genotype led to one band at 730 bp, the bb genotype led to two bands at 654 bp and 76 bp, and the Bb genotype led to three bands at 730 bp, 654 bp, and 76 bp (Fig. 4).

The Minia University ethics committee approved the study protocol.

### Statistical Analysis

(SPSS) v26 statistical software was used to enter and analyze data. A chi-square (Χ²) test was used for categorical data. Independent t-test was used to determine the difference between means in cases and controls. One-way analysis of variance is used to compare between the means of three or more independent groups. P -value < 0.05 was used for statistical significance. MedCalc v20 was used for receiver operating characteristic (ROC) curve.

## Results

This study included 100 patients with rheumatoid arthritis and 50 healthy controls. Among the cases, 18 were male and 82 were female, and among the controls, 14 were male and 36 were female.

**Table 1:** Baseline characteristics of the study participants

	Cases N = 100	Controls N = 50	P	Total
Gender (male/female), n (%)	18/82 (18/82) %	14/36 (28/72) %	0.16	32/118 (21.3/78.7) %
Age (years), M ± SD	45.8 ± 10.01	44.62 ± 9.72	0.49	45.41 ± 9.89
25-(OH)-D (ng/mL), M ± SD	23.58 ± 12	27.74 ± 11	0.04	24.97 ± 11.79
PTH (pg/ ml), M ± SD	40.35 ± 48.98	38.18 ± 26.26	0.77	39.63 ± 42.68
Calcium (mg/dL), M ± SD	8.48 ± 1.17	9.81 ± 1.45	< 0.001	8.93 ± 1.41

M ± SD = mean ± standard deviation, 25-(OH)-D = Serum 25-hydroxy vitamin D

Table 1 shows that the M ± SD serum levels of 25-(OH)-D were 23.58 ± 12 in cases and 27.74 ± 11 in controls. The M ± SD serum intact PTH levels were 40.35 ± 48.98 in cases and 38.18 ± 26.26 in controls. The M ± SD total Ca levels were 8.48 ± 1.17 in cases and 9.81 ± 1.45 in controls.

There was no significant difference in gender, age, and serum intact PTH between the two groups. There was a significant difference between cases and controls regarding the serum level of 25-(OH)-D and Ca (p = 0.04 and < 0.001, respectively).

**Fig. 5:**
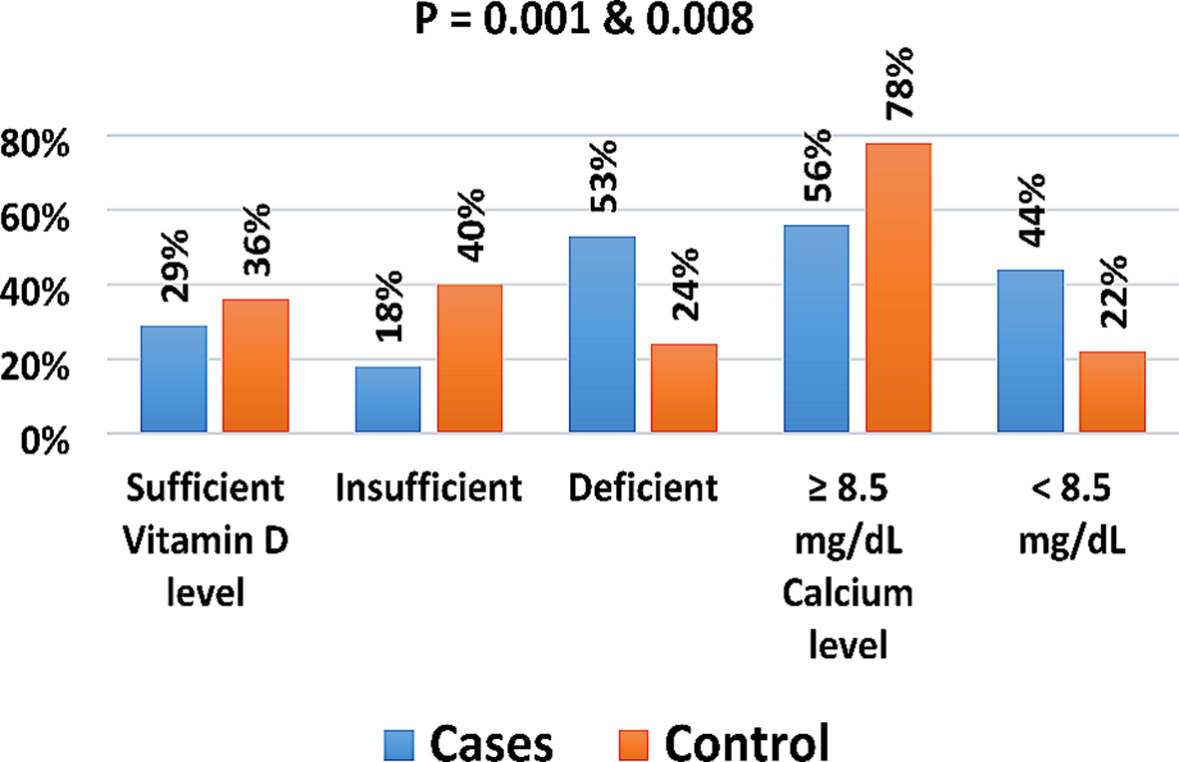
Comparison of serum 25hydroxy vitamin D and Ca levels category between the two groups

Figure 5 shows that the vitamin D levels categories in RA cases and controls were sufficient: insufficient: deficient = 29%:18%:53% and 36%:40%:24%, respectively, (P = 0.001). The calcium levels categories in RA cases and controls were (≥ 8.5 mg/dL): (< 8.5 mg/dL) = 56%:44% and 78%:22%, respectively, (P = 0.008). According to vitamin D and Ca levels there was a significant difference between the two groups.

**Table 2:** **Distribution of the vitamin D receptor gene ApaI and BsmI polymorphisms in cases and controls**

VDR	Cases N = 100		Controls N = 50		X2	P
	N	(%)	N	(%)		
*Apa1 genotype*						
AA	23	23	10	20		
Aa	48	48	24	48	0.24	0.89
aa	29	29	16	32		
A allele	94	47	44	44		
a allele	106	53	56	56		
*BsmI genotype*						
BB	37a	37	22a	44		
Bb	60a	60	16b	32	20.27	< 0.001
bb	3a	3	12b	24		
B allele	134	67	60	60		
b allele	66	33	40	40		

Each subscript letter denotes a subset of group categories whose column proportions do not differ significantly from each other at the 0.05 level

Table 2 showed that no significant difference between cases and controls in terms of the frequencies of the AA, Aa, and aa genotypes (p = 0.89). The frequencies of AA, Aa, and aa genotypes in cases were 23%, 48%, and 29%, respectively, while in controls, they were 20%, 48%, and 32%, respectively. The A allele was present in 47% of cases and 44% of controls, while a allele was found in 53% of cases and 56% of controls. It is noteworthy that having any of the AA, Aa, and aa genotypes does not contribute to RA occurrence.

We observed a significant difference between cases and controls in terms of the frequencies of the genotypes BB, Bb, bb (p < 0.001). The frequencies of BB, Bb, and bb genotypes in cases were 37%, 60%, and 3%, respectively, while in controls, they were 44%, 32%, and 24%, respectively. The B allele was found in 67% of cases and 60% of controls, and the b allele was found in 33% of cases and 40% of controls. The BsmI polymorphism of the VDR gene played a role in the occurrence of RA.

**Table 3:** Vitamin D and calcium levels based on the genotype distribution of ApaI polymorphism

ApaI	25-hydroxy vitamin D (ng/mL) M ± SD	Ca (mg/Dl) M ± SD
AA	22.86 ± 13.44	8.97 ± 0.86
Aa	20.93 ± 11.24	8.38 ± 1.02
aa	28.55 ± 10.83	8.27 ± 1.49
P	0.023	0.064
(AA + Aa)	21.56 ± 11.93	8.57 ± 1
(aa)	28.55 ± 10.83	8.27 ± 1.49
P	0.007	0.25
(aa + Aa)	23.8 ± 11.62	8.34 ± 1.21
(AA)	22.86 ± 13.44	8.97 ± 0.86
P	0.74	0.02

Table 3 displays the M ± SD serum vitamin D levels in AA, Aa, and aa genotypes, which were (22.86 ± 13.44), (20.93 ± 11.24), and (28.55 ± 10.83), respectively, (p = 0.023). The M ± SD serum vitamin D levels in the (AA + Aa) and (aa) genotypes were 21.56 ± 11.93 and 28.55 ± 10.83, respectively, (p = 0.007). The M ± SD serum vitamin D levels in the (aa + Aa) and (AA) genotypes were 23.8 ± 11.62 and 22.86 ± 13.44, respectively, (p = 0.74). There was a contribution of the VDR gene’s ApaI polymorphism to the control of vitamin D levels, with a significantly higher serum vitamin D level associated with the aa genotype.

The M ± SD serum calcium levels among AA, Aa, and aa genotypes were (8.97 ± 0.86), (8.38 ± 1.02), and (8.27 ± 1.49), respectively, (p = 0.064). The M ± SD serum calcium levels in the (AA + Aa) and (aa) genotypes were 8.57 ± 1.0 and 8.27 ± 1.49, respectively, (p = 0.25). The M ± SD serum calcium levels in the (aa + Aa) and (AA) genotypes were 8.34 ± 1.21 and 8.97 ± 0.86, respectively, (p = 0.02). Therefore, a significantly higher serum calcium level is associated with the AA genotype compared to the (aa + Aa) genotypes.

**Table 4:** Vitamin D and calcium levels based on the genotype distribution of BsmI polymorphism

BsmI	25-hydroxy vitamin D (ng/mL) M ± SD	Ca (mg/Dl) M ± SD	
BB	26.13 ± 11.74		8.55 ± 1.17
Bb	21.95 ± 12.15		8.43 ± 1.2
bb	24.97 ± 9.34		8.6 ± 0.56
P	0.25		0.87
(BB + Bb)	23.54 ± 12.11		8.48 ± 1.18
(bb)	24.97 ± 9.34		8.6 ± 0.56
P	0.84		0.86
(bb + Bb)	22.09 ± 12		8.44 ± 1.17
(BB)	26.13 ± 11.74		8.55 ± 1.17
P	0.1		0.64

Table 4 displays the M ± SD serum vitamin D levels among BB, Bb, and bb genotypes, which were (26.13 ± 11.74), (21.95 ± 12.15), and (24.97 ± 9.34), respectively, (p = 0.25). The M ± SD serum vitamin D levels in the (BB + Bb) and (bb) genotypes were 23.54 ± 12.11 and 24.97 ± 9.34, respectively, (p = 0.84). The M ± SD serum vitamin D levels in the (bb + Bb) and (BB) genotypes were 22.09 ± 12 and 26.13 ± 11.74, respectively, (p = 0.1).

The M ± SD serum calcium levels among BB, Bb, and bb genotypes were (8.55 ± 1.17), (8.43 ± 1.2), and (8.6 ± 0.56), respectively, (p = 0.87). The M ± SD serum calcium levels in the (BB + Bb) and (bb) genotypes were 8.48 ± 1.18 and 8.6 ± 0.56, respectively, (p = 0.86). The M ± SD serum calcium levels in the (bb + Bb) and (BB) genotypes were 8.44 ± 1.17 and 8.55 ± 1.17, respectively, (p = 0.64).

Therefore, there is no significant difference between BsmI genotypes regarding 25-hydroxy vitamin D and Ca levels.

**Fig. 6:**
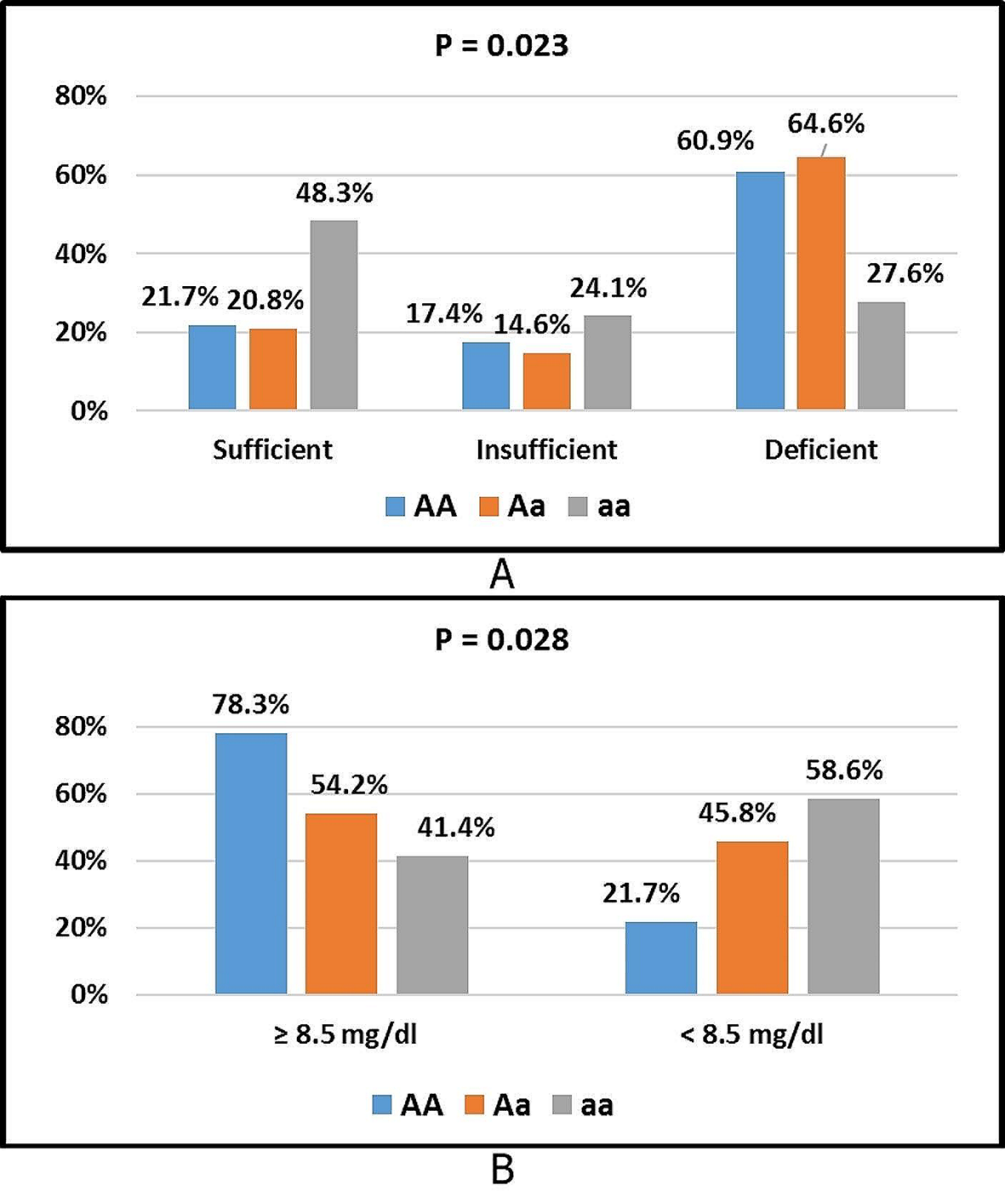
**A** Comparison of serum 25hydroxy Vitamin D levels category among ApaI genotypes. **B** Comparison of serum Ca levels category among ApaI genotypes. Sufficient level = (≥ 30 ng/ml), insufficient level = (20 - < 30 ng/ml), and deficient level = (< 20 ng/ml)

Figure 6(A) shows a significant difference was present in the vitamin D levels category among ApaI genotypes (AA, Aa, and aa) (p = 0.023). The percentages of sufficient vitamin D level category in AA, Aa, and aa genotypes were 21.7%, 20.8%, and 48.3%, respectively. The percentages of insufficient vitamin D level category in AA, Aa, and aa genotypes were 17.4%,14.6%, and 24.1% respectively. The percentages of deficient vitamin D level category in AA, Aa, and aa genotypes were 60.9%, 64.6%, and 27.6%, respectively. A higher percent of sufficient serum vitamin D level category associated with aa genotype and a higher percent of deficient serum vitamin D level category associated with Aa genotype.

Figure 6(B) shows that there was a significant difference in the calcium levels category among ApaI genotypes AA, Aa, and aa (p = 0.028). The percentages of the ≥ 8.5 mg/dL calcium level category in AA, Aa, and aa genotypes were 78.3%, 54.2%, and 41.4%, respectively. The percentages of the < 8.5 mg/dL calcium level category in AA, Aa, and aa genotypes were 21.7%, 45.8%, and 58.6%, respectively. A higher percentage of the ≥ 8.5 mg/dL calcium level category is associated with the AA genotype, and a higher percentage of hypocalcemia is associated with the aa genotype.

**Fig. 7:**
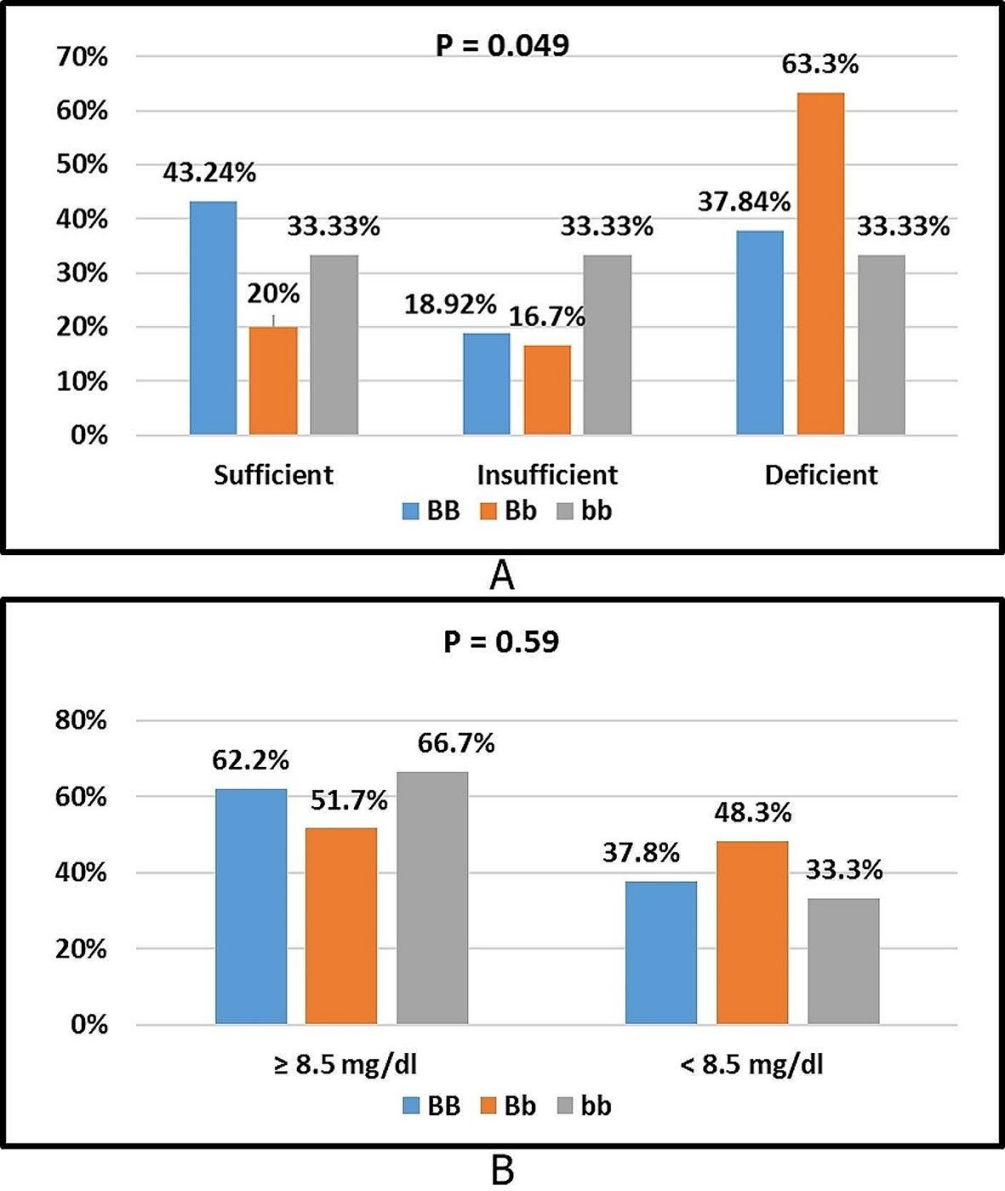
**A** Comparison of serum 25hydroxy vitamin D levels category among BsmI genotypes. **B** Comparison of serum Ca levels category among BsmI genotypes

Figure 7(A) shows a significant difference was present in the vitamin D levels category among BsmI genotypes BB, Bb, and bb (p = 0.049). The percentages of sufficient vitamin D level category in BB, Bb, and bb genotypes were 43.2%, 20%, and 33.3%, respectively. The percentages of insufficient vitamin D level category in BB, Bb, and bb genotypes were 18.9%, 16.7%, and 33.3%, respectively. The percentages of deficient vitamin D level category in BB, Bb, and bb genotypes were 37.8%, 63.3%, and 33.3%, respectively. A higher percentage of sufficient serum vitamin D levels is associated with the BB genotype, and a higher percentage of the deficient serum vitamin D level category is associated with the Bb genotype.

Figure 7(B) shows no significant difference in the Ca levels category among BsmI genotypes BB, Bb, and bb (p = 0.59). The percentages of the ≥ 8.5 mg/dl Ca level category in BB, Bb, and bb genotypes were 62.2%, 51.7%, and 66.7%, respectively. The percentages of the < 8.5 mg/dl Ca level category in BB, Bb, and bb genotypes were 37.8%, 48.3%, and 33.3%, respectively.

**Table 5:** ROC curve analysis for 25-(OH)-D level as a predictor of hypocalcemia

Variable	Cut-off point	AUC	*P*	Sensitivity	Specificity	PPV	NPV	Accuracy
	(ng/ml)			(%)	(%)			(%)
25-(OH)-D level	≤ 19.8	0.53	0.57	58.9	54.5	62.3	51.1	0.135

Table 5 shows that the area under the curve (AUC) was 0.53. The proposed threshold value (cut-off point) was ≤ 19.8 ng/mL with a sensitivity of 58.9% and specificity of 54.5%. The p-value was non-significant (p = 0.57). The curve denotes that the 25-(OH)-D level has not been useful in predicting hypocalcemia.

**Fig. 8:**
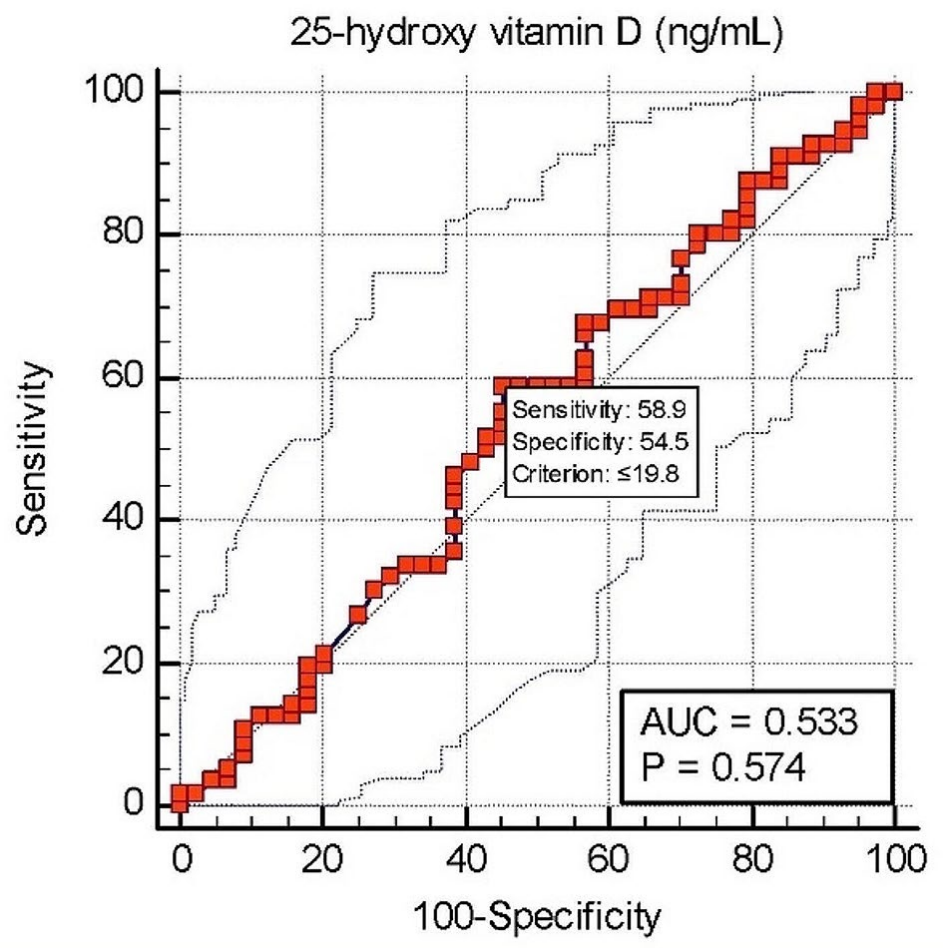
Analysis of ROC curve for 25-(OH)-D level as a predictor of hypocalcemia

Figure 8 shows that the amount of serum vitamin D is not a reliable predictor of hypocalcemia.

## Discussion

We examined the VDR genes ApaI and BsmI polymorphisms, vitamin D levels, and Ca levels in RA patients and healthy control groups. Vitamin D receptor genes ApaI and BsmI polymorphisms were detected using the (RFLP) technique.

There was no significant difference between the cases and controls regarding VDR ApaI gene polymorphism (p = 0.89). However, there was a significant difference between the study groups regarding VDR BsmI gene polymorphism (p < 0.001). This suggests a possible association between VDR BsmI gene polymorphisms and the occurrence of RA disease.

Vitamin D receptor gene could have some effects on RA etiopathology, such as affecting disease onset, and the clinical course of the disease. Rheumatoid arthritis is characterized by bone and joint destruction, therefore it is conceivable that polymorphic genes have a direct effect on vitamin D and Ca metabolism may play a role in RA pathogenesis (Garcia-Lozano et al. 2001).

Studies on RA patients have supported the suggestion that VDR polymorphisms are associated with RA. Specifically, the BsmI polymorphism of the VDR gene is implicated in the development of osteoporosis in RA patients (Lee et al. 2011). In agreement with the current study results, a concluded study suggested that TaqI and ApaI polymorphisms might be modestly implicated in Behcet’s disease (BD) rather than rheumatoid arthritis (RA) pathogenesis. They may serve as possible biomarkers in BD rather than susceptibility genes (Tizaoui et al. 2014). A study confirmed that by comparing RA patients with controls, a direct association between TNFB, BsmI, TaqI, MTHFR (C677T, A1298C), TGFβ1, and ApaI polymorphisms, and RA susceptibility has been demonstrated in this study (Saad et al. 2015). In a study on Behcet’s disease patients, BsmI genotype frequencies for the BD and control groups were BB:Bb:bb = 33.3%:60%:6.7% and 15.6%:44.4%:40%, respectively (P = 0.001). The frequency of the B and b alleles in BD patients was 63.3%:36.7%, respectively, compared to 37.2%:62.2%, respectively, in the control group. The conclusion was that polymorphisms in the VDR gene were associated with susceptibility to BD, which could be related to the immunomodulatory action of vitamin D (Al-Nahas et al. 2017).

An association was reported between VDR BsmI BB genotype and lupus nephritis (P = 0.001). However, no relationship was found between the studied polymorphisms and other clinical manifestations, laboratory profiles of systemic lupus erythematosus (SLE), or disease activity score. Additionally, no relationship was found between VDR BsmI genotypes or alleles and serum 25-hydroxyvitamin D levels among the SLE patients (Mouhamed 2016).

Another study found a significant difference between RA patients and controls in the distribution of VDR-FokI genotype and allele frequencies. FokI polymorphism and the F allele were significantly associated with RA. However, no significant difference was found between RA patients and controls in the distribution of BsmI genotypes (El-Barbary et al. 2015). The detection of VDR in monocytes and activated lymphocytes suggests a role in immunoregulation and raises the possibility that joint inflammation could be influenced by VDR polymorphisms (Bhalla et al. 1983; Provvedini et al. 1983). According to a study, the RA group’s genotypes and alleles for the FokI polymorphism were considerably more different than those of the controls. The FokI F allele and F/F genotype were significantly associated with Behcet’s disease (BD) (P = 0.0003 and 0.002, respectively). Moreover, in the BD group, the FokI polymorphism was linked to the presence of vascular manifestations (P = 0.006). The FokI polymorphism was linked to female gender in RA patients. No relationships were found between the BsmI polymorphism and RA or BD (Karray et al. 2012).

Multiple factors can affect vitamin D levels, including the health of the gut, liver, kidneys, and skin. Dietary and genetic factors also play an important role in regulating vitamin D levels. Because RA is a multisystem disease, it can potentially impact the factors that influence vitamin D levels (Cojocaru et al. 2010), it can affect one or more of these factors, so it can lead to a decrease in vitamin D level. Vitamin D plays a role in autoimmune disorders, and an evidence linking vitamin D as a potential environmental factor implicated in autoimmune diseases continues to accumulate (Al-Nahas et al. 2017).

In the current study, the serum 25-(OH)-D and Ca levels were significantly lower in rheumatoid arthritis patients compared to the healthy control group.

Research results have varied regarding vitamin D levels in RA patients. Studies conducted in many countries have reported that low levels of vitamin D are highly prevalent among RA patients (Furuya et al. 2013; Kerr et al. 2011; Rossini et al. 2010). In contrast, Mukhtar et al. reported that vitamin D levels were found to be sufficient among RA patients, osteoarthritis patients, and controls. There was a non-significant difference among the studied groups in the Pakistani population (Mukhtar et al. 2019).

Calcium levels are determined by multiple hormonal factors (thyroid and parathyroid hormones) and non-hormonal factors, such as nutritional factors, gut integrity, and kidney function. In comparison to healthy controls, in the current study we found that RA patients had considerably decreased serum Ca levels. In agreement with these results, another study showed that The metabolism of phosphorus and calcium is affected in rheumatoid arthritis (RA). Serum levels of calcium and the calcium/phosphorus ratio were decreased, and phosphorus levels were increased, which were highly significant (p < 0.001) in patients with RA compared to healthy controls (Jambale and Halyal 2017). On the contrary, a study revealed no significant difference between RA patients and the healthy control group regarding serum Ca level (Elbassiony et al. 2016).

In the present study, significantly higher serum vitamin D levels were associated with the aa genotype, and significantly higher serum calcium Ca levels were associated with the AA genotype. No significant differences were observed among BsmI polymorphisms regarding raw vitamin D levels. However, a higher percentage of individuals with sufficient serum vitamin D levels were associated with the BB genotype, while a higher percentage of individuals with deficient serum vitamin D levels were associated with the Bb genotype. No significant differences were observed among BsmI polymorphisms regarding Ca levels.

In a study, there was no significant relationship between BsmI gene polymorphism and vitamin D levels; however, vitamin D deficiency was observed to be more common among BB genotype carriers than among Bb genotype carriers. This suggests that the BB VDR genotype is a key factor in causing vitamin D deficiency (Khattab et al. 2022).

Another study revealed that serum 25-(OH)-D levels in beta-thalassemia major patients with the (Ff, ff) and (BB) genotypes were significantly associated with low serum calcium levels (p = 0.08, 0.02, respectively) and low vitamin D levels (p = 0.001, 0.01, respectively). These findings suggest that VDR (FokI, BsmI) gene polymorphisms influence vitamin D levels. Individuals with the (Ff, ff) and BB genotypes were particularly susceptible to low vitamin D levels (Elhoseiny et al. 2016).

Considerable variation exists among ethnicities, with a higher prevalence in populations of European ancestry than in those of Asian ancestry (Alamanos and Drosos 2005; Kochi et al. 2009). Substantial differences in the prevalence of VDR polymorphisms have been observed between races and/or ethnic groups (Cabral et al. 2005; Ting et al. 2008). A remarkable decrease in the risk of RA was observed in Europeans across all genotype models. No significant associations were found in Africans and Arabs (Bagheri-Hosseinabadi et al. 2020). Environmental interactions and ethnicity of the population may be critical in determining the function and expression of VDR (O′ Neill et al. 2013).

The study’s findings could have a significant impact on the progress in the treatment and diagnosis of RA, including:

1. Personalized treatment: VDR genotype could be used to guide treatment decisions, such as selecting disease-modifying antirheumatic drugs (DMARDs) or recommending vitamin D or Ca supplementation.
2. Early diagnosis: VDR genotype could help identify individuals at high risk of developing RA, enabling early intervention and treatment.
3. Monitoring disease progression: VDR genotype could be used to monitor disease progression and predict treatment response.

Further research is necessary to determine the clinical utility of VDR gene polymorphism testing in RA.

## Limitations

First, this study was conducted in one country, and it should be replicated in several countries because different results may occur due to variations in ethnicity. Second, measuring biomarkers took place only once without follow-up to provide longitudinal data.

In summary, this study demonstrated that VDR gene BsmI polymorphism could be a genetic risk marker of RA susceptibility; however, VDR ApaI polymorphism was not associated with RA. Vitamin D and calcium levels were significantly lower in RA patients. ApaI polymorphism of the vitamin D receptor gene was related to vitamin D and calcium levels, while BsmI polymorphism was associated with vitamin D level categories but not with either raw vitamin D level or calcium levels. Serum vitamin D level did not effectively predict hypocalcemia in this study.

## Data Availability

All data generated or analyzed during this study are included in this published article.
